# Overexpression of FAM46A, a Non-canonical Poly(A) Polymerase, Promotes Hemin-Induced Hemoglobinization in K562 Cells

**DOI:** 10.3389/fcell.2020.00414

**Published:** 2020-05-26

**Authors:** Hsi-Hsien Lin, Yu-Ling Lo, Wen-Chih Wang, Kuan-Yeh Huang, Kuan-Yu I, Gin-Wen Chang

**Affiliations:** ^1^Department of Microbiology and Immunology, College of Medicine, Chang Gung University, Taoyuan, Taiwan; ^2^Department of Anatomic Pathology, Chang Gung Memorial Hospital-Linkou, Taoyuan, Taiwan

**Keywords:** cell cycle, differentiation, erythroid cell, nucleotidyltransferase, phosphorylation, poly(A) polymerase

## Abstract

FAM46A belongs to the FAM46 subfamily of the nucleotidyltransferase-fold superfamily and is predicted to be a non-canonical poly(A) polymerase. FAM46A has been linked to several human disorders including retinitis pigmentosa, bone abnormality, cancer, and obesity. However, its molecular and functional characteristics are largely unknown. We herein report that FAM46A is expressed in cells of the hematopoietic system and plays a role in hemin-induced hemoglobinization. FAM46A is a nucleocytoplasmic shuttle protein modified by Tyr-phosphorylation only in the cytosol, where it is closely associated with ER. On the other hand, it is located proximal to the chromatin regions of active transcription in the nucleus. FAM46A is a cell cycle-dependent poly-ubiquitinated short-lived protein degraded mostly by proteasome and its overexpression inhibits cell growth and promotes hemin-induced hemoglobinization in K562 cell. Site-directed mutagenesis experiments confirm the non-canonical poly(A) polymerase activity of FAM46A is essential for enhanced hemin-induced hemoglobinization. In summary, FAM46A is a novel poly(A) polymerase that functions as a critical intracellular modulator of hemoglobinization.

## Introduction

Human chromosome 6 is known to harbor numerous genetic hotspots for retinal diseases such as RP (Small et al., 1992; Stone et al., 1994; Kelsell et al., 1998; Ruiz et al., 1998; Dharmaraj et al., 2000; Kniazeva et al., 2000). One novel candidate disease gene is *C6orf37*, which is mapped within the autosomal recessive RP25 locus on chromosome 6q14 and expressed in the retina (Lagali et al., 2002; Barragan et al., 2008). Interestingly, the full-length *C6orf37* gene product is identical to the FAM46A protein, which is an evolutionary conserved cytosolic protein with multiple putative phosphorylation sites and signaling functions (Lagali et al., 2002; Barragan et al., 2008). In contrast, mouse Fam46a was detected in the developing tooth buds, localized in the nucleus, and interacted with the ZFYVE9 protein (Colland et al., 2004; Etokebe et al., 2009). Hence, it seems that FAM46A is a multifunctional intracellular protein distributed both in the cytosolic and nuclear compartments.

Recently, a novel *FAM46A* nonsense mutation (*FAM46A*^E157*Mhda^) was identified in an ENU-derived BAP014 mutant mouse strain (Diener et al., 2016). As the E157* mutation introduced a pre-mature stop codon after a.a. 156, the homozygous *FAM46A*^E157*Mhda^ mice likely resembled a loss-of-function null mutant. Importantly, these homozygous mutant mice were born with a lower Mendelian ratio and the surviving animals showed a unique phenotype of elevated alkaline phosphatase activities and reduced body size with severe bone abnormalities (Diener et al., 2016). These results indicate that FAM46A is critically involved in embryonic development and adult bone formation and homeostasis. The essential role of FAM46A in bone development was further confirmed recently by Doyard et al. (2018) who identified one nonsense mutation and two different FAM46A homozygous missense variants (p.His^127^Arg and p.Asp^231^Gly) in patients of severe autosomal recessive OI characterized by congenital bowing of the lower limbs. More recently, Watanabe et al. identified FAM46A as a BMP-dependent regulator of pre-placodal ectoderm differentiation in *Xenopus* via specific interaction with Smad1/Smad4 (Watanabe et al., 2018). Additionally, FAM46A was defined as a *trans*-regulator of leptin in a large-scale pQTL analysis of obese individuals (Carayol et al., 2017). In summary, FAM46A is a developmentally important molecule closely associated with diverse pathological disorders including bone abnormality, retinal and metabolic diseases.

Despite these findings, the molecular characteristics and mechanisms of FAM46A remain largely unknown. FAM46A and 3 other human paralogs (FAM46B, C and D) together form the FAM46 family, whose genetic sequences are highly conserved in all available sequenced vertebrates (Kuchta et al., 2009). The FAM46 family has been classified recently as novel members of the nucleotidyltransferase (NTase)-fold superfamily based on the predicted protein domain architecture (Kuchta et al., 2009). The NTase-fold proteins are multifarious and play important roles in biological processes as diverse as RNA polyadenylation and editing, DNA repair, somatic recombination and chromatin remodeling, as well as intracellular signaling and protein activity regulation (Aravind and Koonin, 1999; Rogozin et al., 2003). Interestingly, nearly all known proteins adopting the NTase-fold are able to transfer NMP from NTP to nucleic acid, protein or small molecule containing an acceptor hydroxyl group. More extensive bioinformatics analyses also identified the consensus sequences of the PAP/OAS1 SBD in all FAM46 family members. Importantly, the invariant active-site residues of NTase and essential substrate-binding residues of PAP/OAS1 SBD were all conserved in FAM46 proteins. Based on these criteria, the FAM46 proteins were predicted to be non-canonical poly(A) polymerases (Kuchta et al., 2016). Indeed, Mroczek et al. (2017) recently have provided clear functional evidences of FAM46C working as an active poly(A) polymerase that promotes mRNA stabilization and gene expression.

We herein demonstrate that FAM46A is a cell cycle-dependent nucleocytoplasmic shuttle protein. It is modified by Tyr-phosphorylation only in cytosol but not in nucleus. In addition, we reveal FAM46A is a short-lived protein marked by polyubiquitination and degraded predominantly by proteasomes. We further show that overexpression of FAM46A promotes the hemoglobin synthesis during erythroid differentiation of K562 cells. Importantly, the FAM46A-promoted hemoglobin synthesis is critically dependent on its poly(A) polymerase activity. We conclude that FAM46A plays a role in hemin-induced hemoglobinization as a cell-cycle dependent nucleocytoplasmic non-canonical poly(A) polymerase.

## Materials and Methods

All chemicals and reagents were purchased from Sigma-Aldrich (St. Louis, MO, United States) unless otherwise specified. Antibodies (Abs) used for Western blotting and confocal immunofluorescence analyses include: anti-PARP (Clone A6.4.12) Abs were from Abcam (Cambridge, United Kingdom). Anti-Sp1, anti-actin (C4) mAbs, and Abs to NuMA and Lamin A/C were all from Santa Cruz Biotechnology (Dallas, TX, United States). Anti-cyclin B (clone 18) was from Transduction Laboratories (Lexington, KY, United States). Anti-PDI (MA3-019) mAb was from Thermo Fisher Scientific (Waltham, MA, United States) and anti-transferrin receptor (H68.4) mAb was from Invitrogen. Anti-GAPDH (#60004) was from Proteintech Group, Inc. (Rosemont, IL, United States). mAbs to c-myc epitope (9E10), HA-epitope (12CA5), phospho-Threonine (PTR-8), phospho-Serine (4A4), and AP-1 (clone 100/3) were from Sigma-Aldrich. Anti phospho-Tyrosine (PY20) mAb was from BD Biosciences (San Jose, CA, United States). Abs to Histone H3 (tri-methyl K9) and Histone H3 (acetyl K9) were from Abcam (Cambridge, United Kingdom). Anti-ubiquitin (1510) mAb was from Chemicon International Inc. (Temecula, CA, United States). Rabbit anti-c-Myc agarose conjugate and control rabbit IgG-agarose for IP were purchased from Sigma-Aldrich.

### Cell Culture and RNA Expression Analysis

Murine hematopoietic cell lines of different lineages were cultured as described previously (Lin et al., 1996, 1997). HeLa and K562 cell lines were purchased from American Type Culture Collection (ATCC) (Manassas, VA, United States) and maintained accordingly. Total RNAs were isolated and analyzed from mouse tissues and cell lines of interested as described previously (Lin et al., 1997). Briefly, standard Northern blot analysis was performed using a mouse *Fam46a* cDNA fragment as a probe, while RT-PCR analysis was done using the *Fam46a*-specific primers (Forward primer: 5′-CAAGTGCAGCGGTTGG ACGGCAT-3′; Reverse primer: 5′-CTAAGAGATTGCAG TACT TAAGC-3′) and GAPDH primers (Forward primer: 5′-CGGAG TCAACGGATTTGGTCGTAT-3′; Reverse primer: 5′-AGCCT TCTCCATGGTGGTGAAGAC-3′).

### Construction of the FAM46A Expression Vector

The full-length mouse FAM46A cDNA sequence containing one copy of VNTR was subcloned into the pcDNA3.1-myc C and pEGFP-N1 vectors via appropriate restriction sites to generate the pFAM46A-myc and pFAM46A-EGFP constructs, respectively. The site-directed mutants of FAM46A were generated using the pFAM46A-myc as a template and amplified by PCR with specific primers listed in the Supplementary Table S1. The cDNA fragments of all expression constructs were verified by DNA sequencing. The HA-tagged ubiquitin expression construct was described previously (Cockman et al., 2000).

### Transient Transfection and Subcellular Fractionation

HeLa cells were transfected using the Lipofectamine reagents (Invitrogen) as described previously (Kwakkenbos et al., 2004). Transfection of K562 cells was carried out using the Amaxa Nucleofector^TM^ 2b Device (Amaxa Biosystems, Cologne, Germany). In brief, ∼1 × 10^6^ of K562 cells were mixed with 10 μg of DNA in the “All-in-One” nucleofection buffer in a final volume of 110 μl. DNA-cell mixture was transferred to 2 mm Gene Pulser/MicroPulser Electroporation Cuvettes (Bio-Rad, Hercules, CA, United States). Electroporation was performed using the T016 program. Immediately after electroporation, cells were transferred to flasks and cultured in complete medium.

For subcellular fractionation, cells were thoroughly washed with cold HBSS and re-suspended in cold hypotonic buffer (300 mM sucrose, 5 mM MgCl_2_, 10 mM KCl, 0.1 mM EDTA, 10 mM Tris-Cl, pH 7.8 containing 1 mM sodium orthovanadate, 1 mM AEBSF, 5 mM Levamisole, 10 μg/ml Aprotinin and 1X EDTA-free cOmplete Protease Inhibitor Cocktail). Cells were allowed to swell for 10 min on ice before a final concentration of 0.125% NP-40 was added. Cells were mixed vigorously for 5 sec and centrifuged immediately at 200 *g*, 4°C for 1 min. The supernatant (cytoplasmic fraction) was transferred to a new Eppendorf tube, while the pellets were washed with ice-cold washing buffer (HBSS with the protease inhibitor cocktail) three times. The washed pellets were re-suspended in cold hypertonic buffer (25% glycerol, 5 mM MgCl_2_, 320 mM KCl, 2 mM EDTA, 20 mM Tris-Cl, pH 7.8 containing 1 mM sodium orthovanadate, 1 mM AEBSF, 5 mM Levamisole, 10 μg/ml Aprotinin and 1X EDTA-free cOmplete Protease Inhibitor Cocktail) for 30 min with occasional vigorous mixing, followed by centrifugation at 18,000 *g*, 4°C for 15 min. The supernatant (nuclear fraction) was collected into a new Eppendorf tube. The buffer system in both cytoplasmic and nuclear fractions was readjusted to an isotonic condition containing 100 mM KCl and 20 mM Tris-Cl, pH 7.8.

### Confocal Immunofluorescence Cytochemistry

Confocal immunofluorescence cytochemistry was performed essentially as described previously (Kwakkenbos et al., 2004). Briefly, HeLa and K562 cells transfected with the pFAM46A-EGFP construct were adhered to and cultured on poly-D-lysine coated coverslip (BD Biocoat) for 20 h. Cells were fixed with 4% paraformaldehyde/PBS at room temperature for 20 min. Cell permeabilization was done using PBS/0.5% saponin and then incubated in blocking buffer (2% normal goat serum/0.5% BSA in PBS) for 1 h, followed by incubation with individual primary mAbs against various cytoplasmic and nuclear proteins as indicated. Following extensive washes, cells were stained with Alexa Fluor 647-conjugated 2^nd^ Abs. Cells were then thoroughly washed and the coverslips were placed on the slides and sealed with ProLong Gold (with DAPI) mounting medium (Invitrogen). Confocal images were taken on a Zeiss LSM 510 META confocal microscope. Analysis was performed using LSM510 META software (Zeiss).

### Immunoprecipitation and Western Blotting Analysis

Protein IP was performed essentially as previously described (Kwakkenbos et al., 2004). Briefly, total cell lysate (0.4–1 mg) was pre-cleared by mixing with ∼100 μl of isotype IgG-conjugated agarose for 2 h at 4°C. The mixture was centrifuged at 18,000 *g* for 10 min at 4°C and the supernatant was collected. Approximately 50 μl of 1:1 diluted slurry of Ab-conjugated agarose was added into pre-cleared lysate supernatants and mixed overnight at 4°C on an end-over-end rotator. The agarose beads were then extensively washed with the 1% BSA/cell lysis buffer and the pull-downed proteins were eluted from the beads with the SDS-PAGE sampling buffer and processed for Western blotting analysis.

### Biochemical Analyses of the FAM46A Protein

For protein half-life determination, HeLa cells were seeded in a 35 mm culture dish (2.7 × 10^5^ cells/dish) in complete medium over-night, followed by transfection of the FAM46A-myc construct. Cells were washed 48 h post-transfection and cultured in 2 ml of fresh complete medium containing cycloheximide (20 μg/ml) without or with 1 h pretreatment of indicated protease inhibitors. At desired time points, cells were washed thoroughly and then lysed with cell lysis buffer (60 μl/sample). Whole cell lysates were collected and stored at −80°C until analysis. For the analysis of cell-cycle control of FAM46A protein turnover, transfected HeLa cells were subjected to either double thymidine block for the G1/S phase synchronization or the thymidine-nocodazole block for the G2/M phase synchronization, respectively. In brief, cells were seeded (1.5 × 10^4^ cells/cm^2^) in antibiotics-free complete medium and cultured for over-night before transfection with the FAM46A-myc construct. For the G1/S checkpoint synchronization, cells were washed twice with pre-warmed PBS 24 h post transfection and incubated with pre-warmed fresh complete medium containing thymidine (2 mM) for 20 h. Cells were then rinsed three times with pre-warmed DMEM to remove thymidine, followed by incubation in pre-warmed complete medium for a further 9 h. Cells were washed and cultured in fresh complete medium containing 2 mM thymidine for another 16–18 h. At this point, cells were rinsed thoroughly and replenished with fresh pre-warmed complete medium to release cells from the G1/S block. Cells will start to progress synchronously into G2 phase at ∼6–8 h after release. For the G2/M phase synchronization, cells were transfected as described above and cultured in thymidine (2 mM)-containing complete medium for 24 h. Cells were washed thoroughly to remove thymidine, followed by incubation in complete medium containing 0.1 μg/ml nocodazole for 14–15 h. At the end of incubation, rounded-up cells were collected by tapping dishes several times and aspirated cell suspension into a 50-ml conical tube. Save a portion of these cells separately as the “Time 0” population. Remove nocodazole by washing cells twice with pre-warmed DMEM, then add fresh complete medium to release cells from the G2/M phase synchronously into G1 at around 4 h and into S phase at around 8 h after release. Cellular DNA content was determined by FACS analysis to establish cell cycle stages as described elsewhere.

### Hemin-Induced Erythroid Differentiation of K562 Cells and the Detection of Hemoglobin

Erythroid differentiation of K562 cells was performed as described previously (Baliga et al., 1993). In short, K562 cells (1 × 10^5^ cells/ml) were seeded into 35-mm dishes (4 ml/dish) in complete RPMI medium with or without hemin (50 μM) and cultured for up to 4 days. The extent of hemoglobinization was examined by benzidine staining (Yu et al., 1989). Alternatively, cells were collected at desired time points, washed, and a total of 5 × 10^5^ live cells were used for the colorimetric determination of total hemoglobin using the QuantiChrom Hemoglobin Assay Kit (BioAssay Systems, Hayward, CA, United States) according to the manufacturer’s protocol. When necessary, total cell numbers at desired time points of culture were determined by hemocytometer.

### Statistical Analysis

Experimental data of at least three independent experiments performed in triplicate were quantitatively analyzed using the Student *t*-test by Prism 5 software. The results are shown as means ± standard deviation (SD). In all cases, a probability value of *p*-value < 0.05 was accepted to reject the null hypothesis. The statistical significance of *p* value was set at ^∗^*p* < 0.05, ^∗∗^*p* < 0.01, ^∗∗∗^*p* < 0.001.

## Results

### FAM46A Is a Conserved, Myeloid-Restricted NTase-Like Protein

**FIGURE 1:**
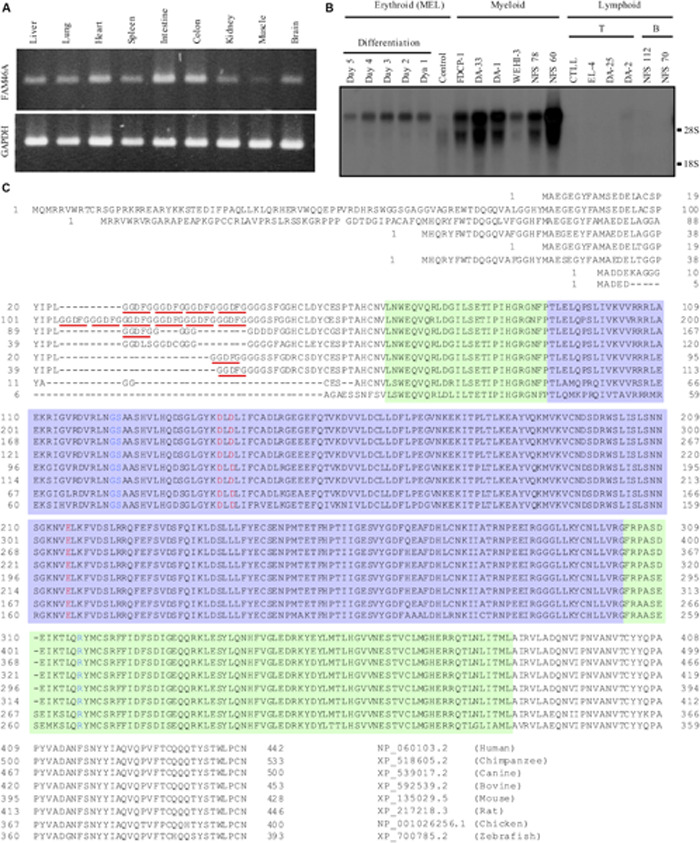
Expressional and structural analyses of FAM46A. **(A)** RT-PCR analysis of *Fam46a* mRNA expression in various mouse tissues as indicated. The strongest *Fam46a* RNA expression was detected in the intestine and colon. Expression of the ubiquitous glyceraldehyde 3-phosphate dehydrogenase (GAPDH) was included as a control (lower panel). **(B)** Northern blot analysis of total RNAs (15 μg/lane) isolated from various mouse hematopoietic cell lines as indicated. *Fam46a* was highly expressed in the myeloid cell lines, but absent in lymphoid cell lines tested. *Fam46a* was induced during the terminal differentiation and maturation of the mouse erythroid cell line (MEL) treated with DMSO. **(C)** Sequence alignment of the FAM46A protein orthologs in different animals as indicated. The protein sequences were aligned for the most sequence identity/homology match using the ClustalW program. The 5-amino acid (GGDFG) repeats encoded by the 15-nucleotide variable number tandem repeat (VNTR) was underlined in red. The predicted core NTase domain and PAP/OAS1 SBD domains are enclosed in the light purple and light green regions, respectively. The invariant active site residues involved in the catalysis of NTase were highlighted in red and the critical NTase substrate binding residues were highlighted in blue.

In a previous study, we identified DD5-3 as one of the differentially expressed cDNAs in the fetal livers of *c-myb* KO mice (Lin et al., 1996, 1997). Subsequent analysis matched DD5-3 sequence to that of the mouse *Fam46a* (*C6orf37*) gene. RT-PCR analysis showed a rather ubiquitous *Fam46a* expression pattern in many mouse tissues, with the strongest signal detected in the intestine and colon (Figure 1A). As c-myb is an important transcription factor for hematopoiesis (Mucenski et al., 1991), *Fam46a* expression in various mouse hematopoietic cell lines was examined. Significant *Fam46a* expression was found in myeloid cells, but almost none if any expression in lymphoid cells. Interestingly, *Fam46a* expression was found to be up-regulated during the terminal differentiation and maturation of the MEL mouse erythroid cell line (Figure 1B). These results suggest that FAM46A might play a functional role in myeloid cells and differentiation of erythroid cells.

Because of the highly conserved nucleotidyltransferase (NTase)-like fold conformation predicted for all FAM46 proteins, FAM46A is thought to have an evolutionary conserved function (Kuchta et al., 2009, 2016). Alignment of different FAM46A protein orthologs indeed showed a striking degree of sequence conservation (Figure 1C). The major difference among the orthologous FAM46A proteins was found at the N-terminal region that contains different copy numbers of a 5-residue (GGDFG) repeat encoded by a 15-bp VNTR (Barragan et al., 2008) (Figure 1C). Following the GGDFG VNTRs is the NTase-fold sequence that is ∼90% identical among the different FAM46A orthologs. Importantly, the predicted active-site and substrate-binding residues involved in the NTase catalytic reaction are 100% identical in all FAM46A orthologs (Figure 1C). Hence, FAM46A is an evolutionary conserved NTase-like protein, very likely an active non-canonical poly(A) polymerase (Kuchta et al., 2016).

### FAM46A Is a Tyr-Phosphorylated Nucleocytoplasmic Shuttle Protein

**FIGURE 2:**
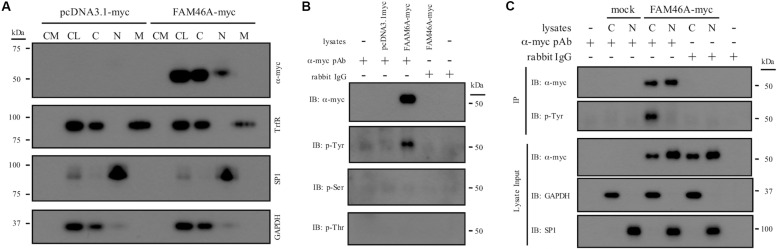
Subcellular distribution and biochemical characterization of the FAM46A protein. **(A)** HeLa cells were transfected with the expression constructs as indicated. Conditioned medium (CM) and total cell lysate (CL) were collected 48 h post-transfection. In addition, protein samples from the cytosolic (C), nuclear (N), and membrane (M) fractions were obtained following the standard subcellular fractionation procedure. Protein samples were separated on SDS-PAGE and subjected to Western blot analysis using specific Abs as indicated. **(B)** Cell lysate of HeLa cells transfected with the indicated expression constructs was immunoprecipitated by polyclonal anti-myc Abs and subjected to Western blot analysis using specific Abs as indicated. Normal rabbit IgGs were included as a control of the polyclonal anti-myc Abs. **(C)** The cytosolic (C) and nuclear (N) protein samples collected from transfected HeLa cells were immunoprecipitated by polyclonal anti-myc Abs and subjected to Western blot analysis using specific Abs as indicated. The top three panels were the lysate input controls. The data shown were the representatives of three separate experiments.

To investigate the molecular characteristics of FAM46A, we first examined its subcellular localization and biochemical properties. Protein samples from HeLa and K562 cells transfected with a myc-tagged mouse FAM46A expression construct were analyzed by Western blotting (WB). FAM46A was readily detected in total cell lysate but not in conditioned medium, suggesting it is not a secreted protein. Analyses of different subcellular constituents identified FAM46A in the cytosolic and nuclear, but not membranous fractions (Figure 2A and Supplementary Figure S1). Of note, FAM46A was detected predominantly in the cytosolic compartment with minimal nuclear allocation. These results indicate that FAM46A is an intracellular protein distributed differentially in both cytosolic and nuclear compartments.

Proteins that shuttle between cytosol and nucleus are usually modulated by unique protein modification such as phosphorylation. As many putative phosphorylation sites were predicted in FAM46A, its phosphorylation status was further examined. IP and WB analyses showed that FAM46A is specifically modified by phosphorylation at Tyrosine, but not Serine or Threonine residues (Figure 2B). Most intriguingly, Tyr-phosphorylation was detected exclusively in the cytosolic but not nuclear FAM46A protein of transfected HeLa (Figure 2C) and K562 cells (data not shown).

**FIGURE 3:**
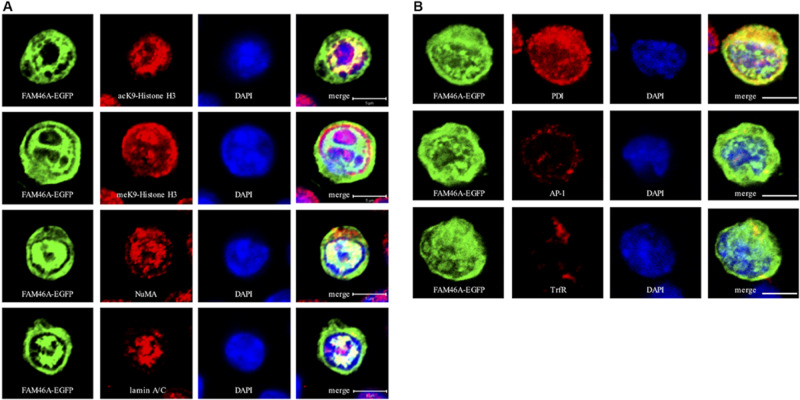
Confocal analysis of the subcellular localization of FAM46A in transfected K562 cells. Cells were transfected with an expression construct encoding the FAM46A-EGFP protein, which was detected in the nuclear **(A)** and cytoplasmic **(B)** compartments. Within the nucleus **(A)**, FAM46A was predominantly confined in the chromatin domains more accessible to the transcription complexes (as indicated by the acK9-Histone H3 staining) and were mostly excluded from the regions with condensed chromatin structures (as indicated by the meK9-Histone H3 staining). In addition, significant co-localization of FAM46A with NuMA and lamin A/C was observed. DAPI staining defined the morphology of nuclei. Scale bar: 5 μm. Within the cytoplasm **(B)**, the majority of FAM46A was found to co-localize with the endoplasmic reticulum marker, protein disulfide-isomerase (PDI). By contrast, co-localization of FAM46A with trans-Golgi network marker γ-adaptin (AP-1) and early endosome marker transferrin receptor (TrfR) is relatively low. DAPI staining defines the morphology of nuclei. Scale bar: 5 μm.

To further define the subcellular localization of FAM46A, confocal immunofluorescence analysis of cells expressing the FAM46A-EGFP protein was performed in combination with specific cytoplasmic and nuclear markers. In the nucleus, FAM46A was found predominantly in the chromatin regions more accessible to the transcription complexes as marked by the acK9-Histone H3 staining. Consequently, FAM46A was mostly excluded from the condensed chromatin domains indicated by the meK9-Histone H3 staining (Figure 3A and Supplementary Figure S2A). In addition, significant co-localization of FAM46A with the nuclear matrix marker NuMA and the peripheral nuclear lamina marker lamin A/C was readily observed. In the cytoplasm, FAM46A seemed to be co-localized mainly with protein disulfide-isomerase (PDI), the ER marker. By contrast, very little co-localization of FAM46A with trans-Golgi network marker γ-1-adaptin (AP-1) and early endosome marker transferrin receptor (TrfR) was noted (Figure 3B and Supplementary Figure S2B). Taken together, we conclude that FAM46A is a nucleocytoplasmic shuttle protein that is Tyr-phosphorylated in the cytosol, but de-phosphorylated in the nucleus. The nuclear FAM46A is located at nuclear matrix and peripheral nuclear lamina, and proximal to the unwound chromatin regions whereas the cytoplasmic FAM46A appears to associate predominantly with ER.

### FAM46A Is a Short-Lived, Cell Cycle-Dependent Protein

**FIGURE 4:**
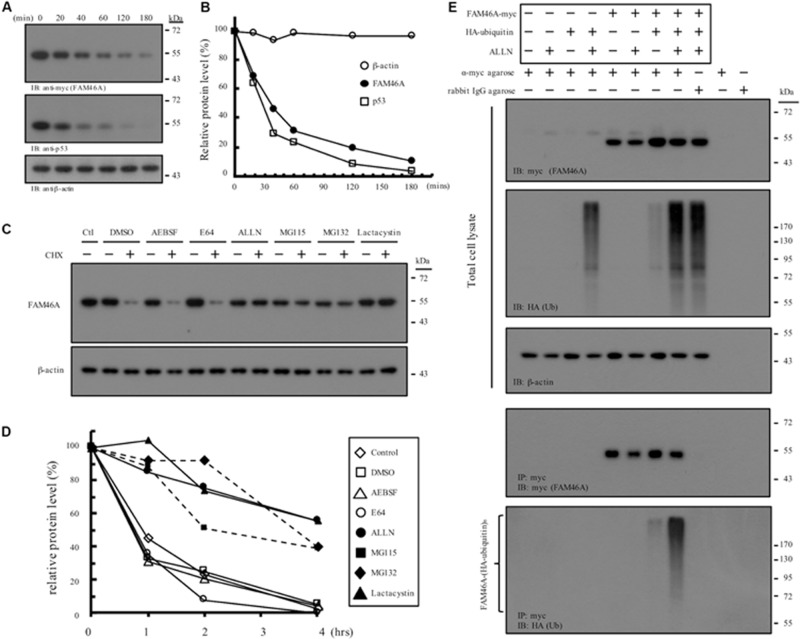
Molecular characterization of FAM46A protein stability and turnover. **(A,B)** FAM46A is a short-lived protein. **(A)** Western blot analysis of the FAM46A-myc protein in transfected HeLa cells treated with cycloheximide (20 μg/ml). Total cell lysates were collected at different time points (minutes) shown at the top of the panels. Blots were probed with Abs to detect FAM46A, p53 and β-actin as indicated. **(B)** The estimation of the half-life of the indicated proteins. The intensity of the protein bands depicted in **(A)** was determined by the densitometer and all values were calculated as a percentage of the starting protein level at time 0. Protein half-life was determined by calculating the time required to achieve 50% of the initial protein level. The data shown were the representatives of three independent experiments with similar results. **(C,D)** FAM46A protein expression was stabilized in the presence of proteasome inhibitors. **(C)** Western blot analysis of the FAM46A protein expression levels in transfected HeLa cells treated with various protease/proteasome inhibitors in the presence or absence of cycloheximide (CHX, 20 μg/ml) for 4 h. For CHX treatment, CHX was added 1 h after the addition of protease/proteasome inhibitors. Blots were probed with the anti-myc Ab to detect FAM46A. Expression of β-actin was included as a loading control. **(D)** Changes in half-life of the FAM46A protein following treatment of protease/proteasome inhibitors. All values were calculated as a percentage of the starting protein level at time 0. **(E)** FAM46A is modified by poly-ubiquitylation. HeLa cells were co-transfected with the FAM46A-myc and HA-tagged ubiquitin expression constructs as indicated. Cells were treated with either DMSO (–) or 100 μM ALLN (+) for 16 h. Whole cell lysates were subjected to immunoprecipitation using rabbit anti-c-myc agarose conjugate. Blots were probed with the anti-myc and anti-HA mAbs to detect FAM46A and ubiquitylated proteins, respectively. Expression of β-actin was included as a loading control.

Nucleocytoplasmic shuttle proteins are normally regulated at multiple levels, including unique post-translational modification, dynamic intracellular trafficking, and tightly controlled protein turnover. We hence examined the stability regulation of FAM46A protein. WB analysis of cycloheximide-treated HeLa cells expressing FAM46A-myc showed that FAM46A protein has a relatively short half-life (*t*_1__/__2_ ≈ 35 min), which is comparable to that of the well-known tumor suppressor p53 protein (*t*_1__/__2_ ≈ 20–25 min) (Figures 4A,B). Moreover, the expression of FAM46A protein was stabilized in the presence of specific proteasome inhibitors, but not other protease inhibitors (Figures 4C,D and Supplementary Figure S3). Finally, IP analysis of cells co-transfected with FAM46A-myc and HA-tagged ubiquitin constructs revealed that FAM46A is modified by poly-ubiquitination (Figure 4E and Supplementary Figure S4).

**FIGURE 5:**
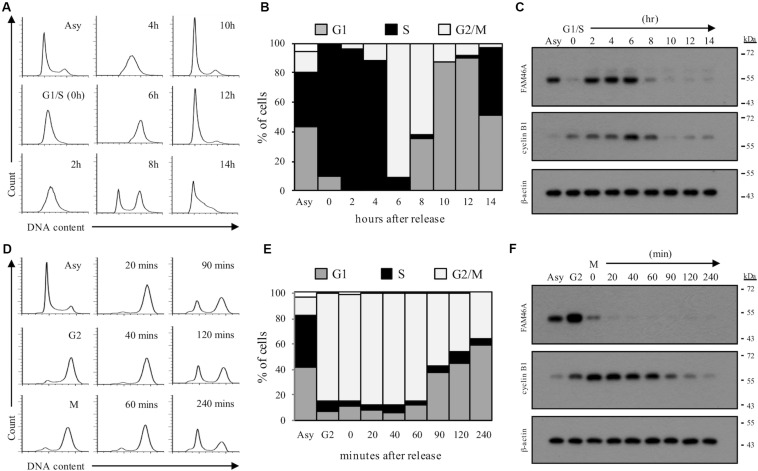
Cell cycle-regulated expression of FAM46A protein. FAM46A-myc transfected HeLa cells were synchronized at the G1/S boundary by double thymidine block **(A–C)** or at the G2/M boundary by thymidine-nocodazole block **(D–F)**, and released at various time points as indicated. Cell cycle progression was monitored by the FACS analysis of cellular DNA contents **(A,D)**. The profiles of synchronous peaks were fitted with Dean-Jett-Fox model using FlowJo software. Percentage of cells in the G1, S, and G2/M phases was presented **(B,E)**. Changes in protein levels of FAM46A and cyclin B1 during cell cycle progression were revealed using Western blot analysis **(C,F)**. Equal amounts (30 μg) of whole cell lysates collected from synchronized cells were loaded in each lane. Expression of β-actin was included as a control. Data shown were representatives of three independent experiments.

We next asked if the rapid FAM46A protein turnover is cell cycle-regulated. Transfected HeLa cells were synchronized at the G1/S and G2/M boundary by double-thymidine block and thymidine-nocodazole block, respectively. Cells were then released from the specific cell cycle stages for analysis. Interestingly, FAM46A was shown to be weakly expressed at the G1 phase then becomes highly expressed at the S and G2 phases, followed by a sharp decrease at the M phase (Figure 5). Taken together, these results indicate that FAM46A is a cell cycle-dependent short-lived nucleocytoplasmic protein, which is polyubiquitinated and processed mostly through proteasome degradation.

### FAM46A Inhibits Cell Growth and Induces Hemoglobinization

**FIGURE 6:**
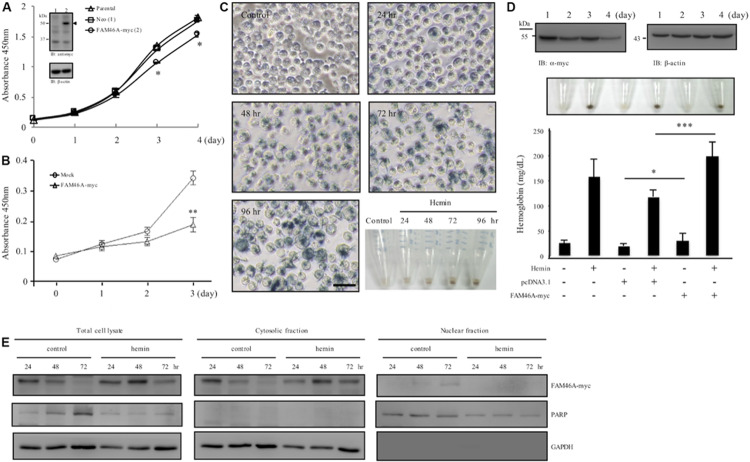
FAM46A inhibits cell growth and promotes hemin-induced hemoglobinization. **(A)** Comparison of the cell growth curve of different HeLa cell populations as indicated. Inset showed the WB analysis of FAM46A protein (arrowhead) expression in HeLa cells stably expressing FAM46A (lane 2). The Neo-control HeLa cell (lane 1) was included as a control (*n* = 3, mean ± SD; ^∗^*p* < 0.05). **(B)** Comparison of the cell growth curve of K562 cells either mock-transfected or transiently transfected with the FAM46A-myc construct (*n* = 4, mean ± SD; ^∗∗^*p* < 0.01). **(C,D)** Analysis of hemin-induced erythroid differentiation of K562 cells. Cells were treated with hemin (50 μM) and cultured for up to 4 days, followed by benzidine staining as described in section “Materials and Methods” **(C)**. Cells were observed under microscopy and photographs were taken to show stain-positive cells representing differentiated cells (blue). Scale: 50 μm. The lower right panel shows the cell pellets collected at different time points as indicated. **(D)** The effect of FAM46A expression in K562 cell differentiation. Top panel: WB analysis of FAM46A expression in transiently transfected K562 cells. FAM46A was detected by the anti-myc Ab, while β-actin was used as a loading control. Middle panel: photographs of cell pellets showing stain-positive differentiated cells (blue). Lower panel: Analysis of hemoglobin production in transfected K562 cells treated with or without hemin at day 3 post transfection (*n* = 3, mean ± SD; ^∗^*p* < 0.05, ^∗∗∗^*p* < 0.001). **(E)** Western blot analysis of the subcellular distribution of the FAM46A protein in K562 cells treated with or without hemin at different time points as indicated. Expression of GAPDH and PARP is used as a marker for the cytosolic and nuclear fractions, respectively.

While investigating molecular characteristics of FAM46A in transiently transfected HeLa cells, apparent cell growth retardation was noticed (Supplementary Figure S5). To further explore this phenotype, we generated stable HeLa cells over-expressing FAM46A-myc. As expected, significant cell growth inhibition was noted in FAM46A-expressing cells in comparison to the parental cells and Neo-control cells (Figure 6A). As our earlier results indicated a possible role of FAM46A in hemin-induced hemoglobinization, we hence investigated the effect of FAM46A expression in regulating cell proliferation of K562 erythroid leukemia cells. Consistent with the results of HeLa cells, FAM46A expression also retarded cell proliferation in transiently transfected K562 cells (Figure 6B). Impeded cell growth is a characteristic of cell differentiation and erythroid differentiation can be efficiently induced in K562 cells in the presence of hemin (Figure 6C). Therefore, we looked into the effect of FAM46A expression in hemin-induced K562 erythroid differentiation by determining the production of hemoglobin, an erythropoiesis marker (Wojda et al., 2002). At steady states, the FAM46A protein level in transiently transfected K562 cells remained stable for up to 2 days post-transfection and declined gradually afterward. At day 4 post-transfection, only approximately 20–30% of FAM46A protein was detected compared to day 1 (Figure 6D). This result is consistent with the finding that FAM46A is a short-lived protein. When compared to control and mock-transfected cells, significantly enhanced hemoglobin production was detected in FAM46A-expressing cells, suggesting that forced FAM46A expression in K562 cells promotes hemin-induced erythroid differentiation. Moreover, a higher hemoglobin level was also found in FAM46A-expressing K562 cells in the absence of hemin (Figure 6D), further supporting a role for FAM46A as an inducer of erythroid differentiation.

As FAM46A was shown as a short-lived nucleocytoplasmic NTase-like protein, we wanted to know how these characteristics are linked to its role as erythroid differentiation promoter. Hence, the expression patterns of FAM46A protein in the cytosolic and nuclear compartments during K562 erythroid differentiation were compared. We detected more FAM46A protein at days 2 and 3 in total cell lysates of hemin-treated cells in comparison to untreated controls, suggesting reduced degradation (Figure 6E). Furthermore, the more stable FAM46A protein was identified mostly in the cytosolic fraction, while little if any FAM46A is detected in the nuclear fraction of the hemin-treated cells. By contrast, nuclear FAM46A protein was readily detected in untreated control cells at day 3 (Figure 6E). Hence, cytosolic FAM46A protein seems to be more stable and remains longer in the presence of hemin.

### FAM46A Over-Expression Promotes Erythroid Differentiation in an Poly(A) Polymerase Activity-Dependent Manner

**FIGURE 7:**
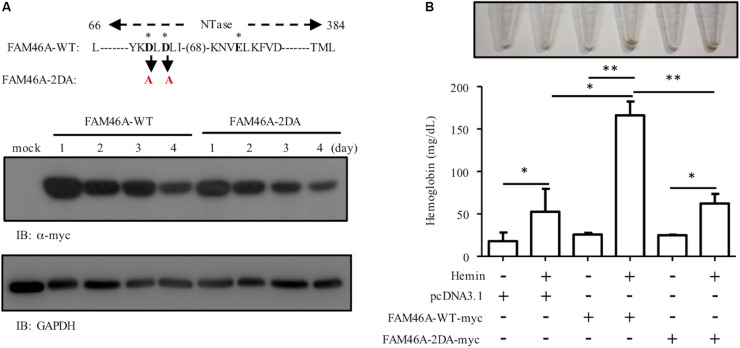
The poly(A) polymerase activity is necessary for the role of FAM46A in promoting erythroid differentiation. **(A)** WB analysis of the expression of FAM46A-WT and FAM46A-2DA in transfected K562 cells at different time points as indicated. The FAM46A protein sequence of interest is listed for the WT molecule, while the specific mutated residues are highlighted in red (and denoted in black arrow). The three asterisks represent the conserved NTase active site residues. **(B)** The effect of the FAM46A-2DA mutant variant on the modulation of K562 erythroid differentiation. Hemoglobin production is analyzed in K562 cells transfected with indicated FAM46A expression constructs and treated with or without hemin for 3 days (*n* = 3, mean ± SD; ^∗^*p* < 0.05, ^∗∗^*p* < 0.01, ns, non-significant).

To investigate the role of the poly(A) polymerase activity of FAM46A in promoting erythroid differentiation, we generated the mutant FAM46A variant, FAM46A-2DA, in which two highly conserved active-site Asp residues of NTase were changed to Ala. The FAM46A-2DA variant is expected to be an enzymatically inactive protein as the same point mutations in FAM46C have been shown to efficiently inhibit the non-canonical poly(A) polymerase activity (Mroczek et al., 2017). The expression level of FAM46A-2DA was comparable to that of WT FAM46A, suggesting the D to A mutations did not cause any significant conformational instability (Figure 7A). Nevertheless, the hemoglobin production of K562 cells expressing FAM46A-2DA was much reduced in comparison to that of FAM46A-WT expressing cells, and was rather similar to that of mock-transfected cells (Figure 7B). This result strongly suggests that the poly(A) polymerase activity is critical for FAM46A as an inducer of erythroid differentiation.

## Discussion

The FAM46 proteins are the least studied members of the NTase-fold superfamily (Kuchta et al., 2016). Previous genetic and proteomic analyses in mutant mice and human patients as well as *in vivo* manipulation in *Xenopus* have provided definite evidences for a role of FAM46A in metabolic pathways, embryonic and bone development, as well as cancer and retinal diseases (Lagali et al., 2002; Cui et al., 2006; Barragan et al., 2008; Etokebe et al., 2015b; Diener et al., 2016; Carayol et al., 2017; Doyard et al., 2018; Watanabe et al., 2018). We demonstrate herein for the first time the presence and function of FAM46A in the hematopoietic system, especially hemin-induced hemoglobinization (Figures 1, 6). FAM46A has been reported to interact with distinct intracellular proteins including ZFYVE9 and Smad1/4 (Colland et al., 2004; Watanabe et al., 2018). Interestingly, ZFYVE9 is an important cytosolic adaptor protein linking the type-I TGF-β receptors and Smad2/3 (Lin et al., 2004), while Smad 1 is a critical BMP-induced signal transducer that traffics to nucleus as a transcription factor (Blank and Karlsson, 2015). Both TGF-β and BMP belong to the TGF-β superfamily that plays an important role in diverse developmental and physiological processes including erythropoiesis as well as bone development (Wu and Hill, 2009; Weiss and Attisano, 2013). Therefore, based on our present results along with the clear association of FAM46A with the abnormal bone phenotypes in the *FAM46A*^E157*Mhda^ mice and OI patients, it is reasonable to suggest that FAM46A is likely a critical intracellular modulator of the TGF-β superfamily.

The differential subcellular distribution pattern and cytosol-specific Tyr phosphorylation of FAM46A protein (Figures 2, 3) are in line with the expression and functional regulation of many nucleocytoplasmic shuttle proteins (Cartwright and Helin, 2000; Gama-Carvalho and Carmo-Fonseca, 2001). Hence, it is envisioned that differential Tyr phosphorylation of FAM46A protein is a decisive factor in the regulation of its stability, function, and distribution in the cytosolic and nuclear compartments by interacting with its specific binding partner(s). Thus, it will be of interest to identify the interacting molecules and mechanisms delineating the close proximity of the nuclear FAM46A to the chromatin region of active transcription and the ER-preferred localization of its cytosolic counterpart (Figure 3). Regarding the ER association of cytosolic FAM46A, it is worth mentioning that FAM46A is unlikely to be a secretive or membrane protein due to the lack of a signal peptide and transmembrane domain. Hence its co-localization with the ER lumen PDI enzyme probably reflects its close association with the cytosolic face of ER. With this in mind, it is intriguing to note that the majority of mRNA substrates polyadenylated by FAM46C were found to encode proteins that are targeted to the ER/Golgi apparatus (Mroczek et al., 2017). Thus, there might also be a locational-functional link for cytosolic FAM46A such that the ER-associated FAM46A binds and polyadenylates specific mRNA transcripts that encode proteins designated for ER. It is similarly intriguing to note its modification by poly-ubiquitination, leading to a relatively short half-life by proteasome degradation (Figure 4). In the future, the identification of the exact Tyr residue(s) and the kinase(s)/phosphatase(s) involved in FAM46A phosphorylation will be of importance to elucidate these unique characteristics.

It is generally known that trafficking of nucleocytoplasmic shuttle proteins into and out of the nucleus required specific signals and transport receptor/adaptor molecules (Cartwright and Helin, 2000; Gama-Carvalho and Carmo-Fonseca, 2001). Furthermore, such nuclear protein transport systems are usually critical in many aspects of cell physiology, including cell cycle control. Thus, it is not surprising to identify the cell cycle-dependent regulation of FAM46A protein expression (Figure 5). It is interesting, however, to note that the dynamic FAM46A turnover during cell cycle progression is very similar to that of cyclin A, which is a key regulatory protein in the cell cycle S and the G2/M phases (Henglein et al., 1994; Huet et al., 1996). How the FAM46A function is linked to the cell cycle-regulated protein expression remains to be investigated.

Our findings of the role of FAM46A in modulating erythroid cell growth and differentiation are in line with that of FAM46C (Mroczek et al., 2017). As mentioned above, both FAM46A and FAM46C paralogs share a highly conserved NTase-like sequence homology and are predicted to be putative non-canonical poly(A) polymerases (Kuchta et al., 2016). FAM46C is a tumor suppressor whose gene is frequently mutated/deleted in multiple myeloma (Mroczek et al., 2017; Zhu et al., 2017). Recently, FAM46C was shown to indeed function as an active non-canonical poly(A) polymerase capable of enhancing mRNA polyadenylation, stabilization, and gene expression (Mroczek et al., 2017). While we did not demonstrate directly the poly(A) polymerase activity for FAM46A, the results from the FAM46A-2DA mutant have provided a highly indicative evidence. The two Asp residues are predicted to be part of the essential NTase active sites and the same D to A mutation in FAM46C has been shown to inhibit the enzyme activity. Hence, it can be summarized that both FAM46A and FAM46C are bona fide non-canonical poly(A) polymerases. Most importantly, FAM46C-deficient mice were found to have increased RBC counts and significantly lower hemoglobin levels (Mroczek et al., 2017), indicating a defect in erythroid cell growth and differentiation. Judging from these similar phenotypes, it is very likely both FAM46A and FAM46C share similar functions as critical non-canonical poly(A) polymerases in the hematopoietic system.

Nevertheless, both FAM46 proteins clearly also have distinct cell/tissue-specific functions as the FAM46C KO mice did not seem to have any bone abnormality as those of the FAM46A mutant animals and OI patients (Mroczek et al., 2017). The future identification of the mRNA targets of FAM46A will be instrumental in revealing the molecular mechanisms involved in modulating hemin-induced hemoglobinization and bone development by FAM46A. Finally, FAM46A is hallmarked by the presence of the polymorphic VNTRs in its second exon (Barragan et al., 2008) (Figure 1C). Different types of VNTRs have been identified in the genome and it is generally thought that these minisatellite repeats might modulate the biological functions of the VNTR-containing protein variants (Nakamura et al., 1998). The FAM46A-specific VNTR consists of a consensus 15-bp sequence encoding a Glycine-rich 5-residue fragment, and the polymorphic FAM46A VNTRs have been associated with non-small cell lung cancer and susceptibility to tuberculosis as well as osteoarthritis in Croatian populations (Etokebe et al., 2014, 2015a,b). By contrast, no apparent association was found between the FAM46A VNTR polymorphism and the risk of colorectal cancer in a group of Chinese patients (Cui et al., 2006). Because the FAM46A protein investigated in the present study contains only one VNTR, it cannot be ruled out that more functional complexities might be manifested by FAM46A protein variants containing more VNTRs. In conclusion, the FAM46A non-canonical poly(A) polymerase represents a previously unappreciated molecular modulator and potential therapeutic target for specific bone, retinal, and hematopoietic disorders.

## Data Availability Statement

All datasets presented in this study are included in the article/Supplementary Material.

## Author Contributions

H-HL, Y-LL, W-CW, K-YH, K-YI, and G-WC performed the experiments and analyzed the data. H-HL and G-WC wrote the manuscript.

## Conflict of Interest

The authors declare that the research was conducted in the absence of any commercial or financial relationships that could be construed as a potential conflict of interest.

## Abbreviations

BMP: bone morphogenetic protein
ENU: *N*-ethyl -*N*-nitrosourea
ER: endoplasmic reticulum
FAM46A: family-with-sequence-similarity 46 member A
IP: immunoprecipitation
NMP: nucleoside monophosphate
NTP: nucleoside triphosphate
NTase: nucleotidyltransferase
OAS1: 2 ′ -5 ′ -oligoadenylate synthetase 1
OI: osteogenesis imperfecta
PAP: poly(A) polymerase
pQTL: protein quantitative trait locus
RP: retinitis pigmentosa
SBD: substrate-binding domain
TGF: transforming growth factor
VNTRs: variable number tandem repeats
WT: wild-type
ZFYVE9: zinc finger FYVE domain-containing 9.
