# Supplementary material for: Overexpression of FAM46A, a Non-canonical Poly(A) Polymerase, Promotes Hemin-Induced Hemoglobinization in K562 Cells
Source: Front Cell Dev Biol. 2020 May 26;8:414. doi: 10.3389/fcell.2020.00414 (PMC7264091; doi:10.3389/fcell.2020.00414)
Supplement: Supplementary file 1 [file Data_Sheet_1.PDF]

Supplemental information for

Overexpression of FAM46A, a non-canonical poly(A) polymerase, promotes hemin-induced hemoglobinization in K562 cells

Hsi-Hsien Lin[1,2,*], Yu-Ling Lo[1], Wen-Chih Wang[1], Kuan-Yeh Huang[1], Kuan-Yu I[1], Gin-Wen Chang[1]

[1]Department of Microbiology and Immunology, College of Medicine, Chang Gung University, Taoyuan, Taiwan. [2]Department of Anatomic Pathology, Chang Gung Memorial Hospital-Linkou, Taoyuan, Taiwan.

*** Correspondence:** Dr. Hsi-Hsien Lin, Department of Microbiology and Immunology, College of Medicine, Chang Gung University, Taoyuan, Taiwan. Email: hhlin@mail.cgu.edu.tw

Running title: FAM46A in hemin-induced hemoglobinization

Supplementary Table 1. Sequences of primers used to generate FAM46A mutants

FAM46A-2DA -5	5'-GGC TAC AAG GCA CTG GCA CTC ATC TTC TGC GCT-3'
FAM46A-2DA -3	5'-GAA GAT GAG TGC CAG TGC CTT GTA GCC TAG GCC-3'

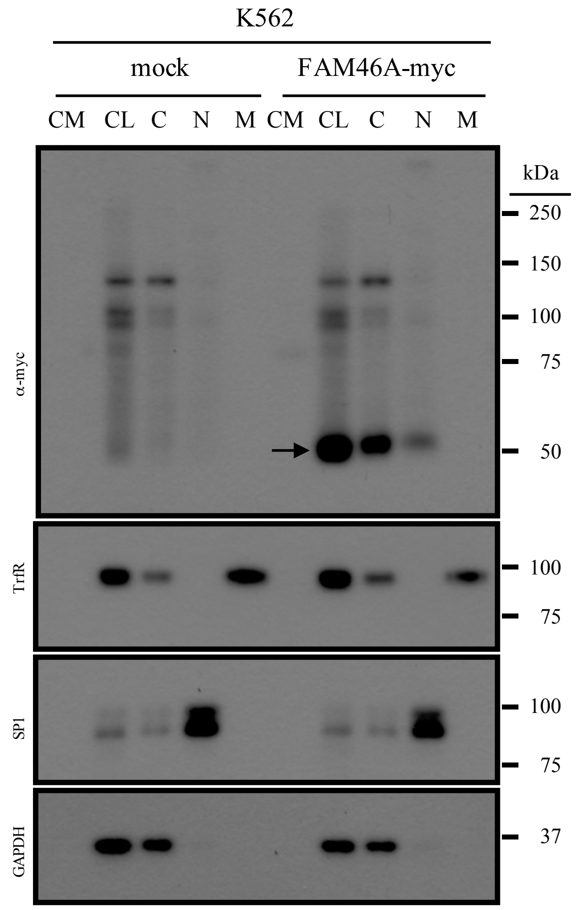

Supplemental Fig. 1 Subcellular distribution of the FAM46A protein. (A) K562 cells were transfected with the expression constructs as indicated. Conditioned medium (CM) and total cell lysate (CL) were collected 48 hr post-transfection. In addition, protein samples from the cytosolic (C), nuclear (N) and membrane (M) fractions were obtained following the standard subcellular fractionation procedure. Protein samples were separated on SDS-PAGE and subjected to Western blot analysis using specific Abs as indicated.

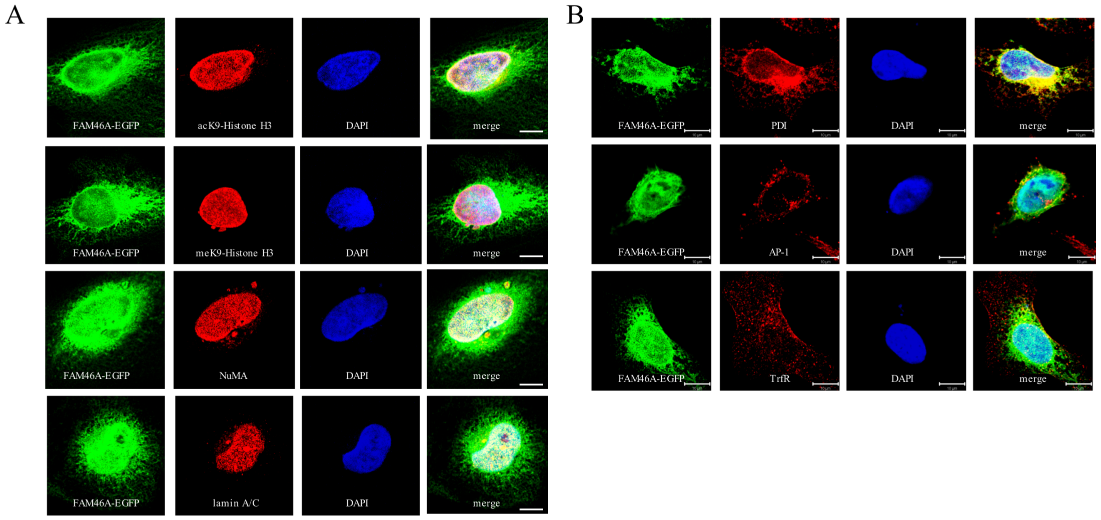

Supplemental Fig. 2 Confocal analysis of the subcellular localization of FAM46A in transfected HeLa cells. Cells were transfected with an expression construct encoding the FAM46A-EGFP protein, which was detected in the nuclear (A) and cytoplasmic (B) compartments. Within the nucleus (A), FAM46A was predominantly confined in the chromatin domains more accessible to the transcription complexes (as indicated by the acK9-Histone H3 staining) and were mostly excluded from the regions with condensed chromatin structures (as indicated by the meK9-Histone H3 staining). In addition, significant co-localization of FAM46A with NuMA and lamin A/C was observed. DAPI staining defined the morphology of nuclei. Scale bar: 10 μm. Within the cytoplasm (B), the majority of FAM46A was found to co-localize with the endoplasmic reticulum marker, protein disulphide-isomerase (PDI). By contrast, co-localization of FAM46A with trans-Golgi network marker γ-adaptin (AP-1) and early endosome marker transferrin receptor (TrfR) is relatively low. DAPI staining defines the morphology of nuclei. Scale bar: 10 μm.

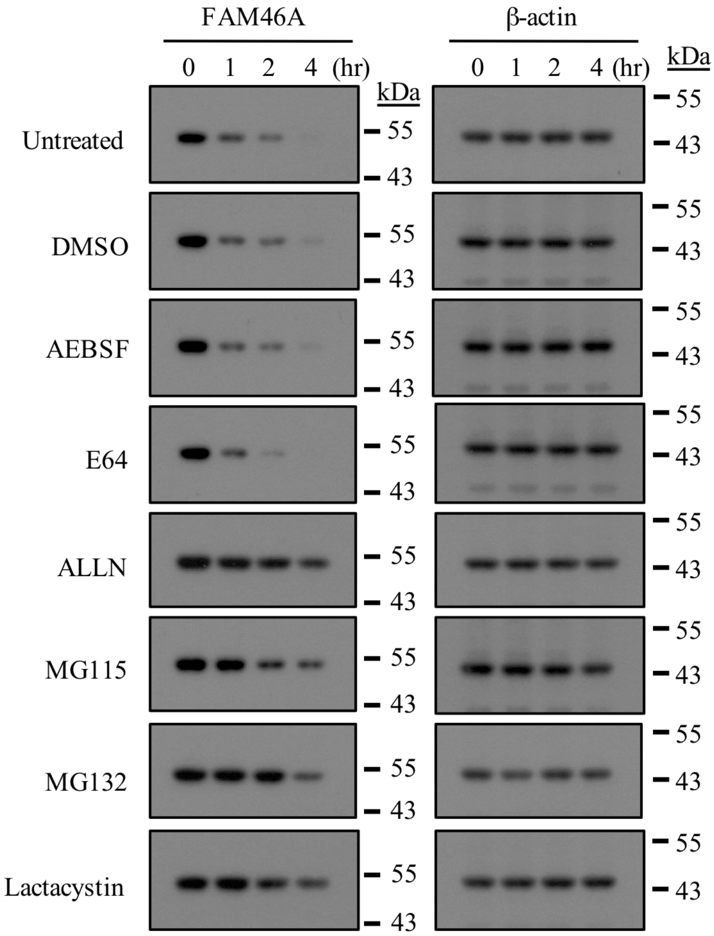

Supplemental Fig. 3 FAM46A protein is degraded by the proteasome-mediated pathway. HeLa cells were transiently transfected with the pFAM46A-myc. Forty-eight hours post-transfection, cells were treated without or with various protease/proteasome inhibitors for 1 hour prior to the addition of cycloheximide (20 μg/ml) for the time period indicated at the top. The expression levels of FAM46A (left panel) and β-actin (right panel) proteins were revealed by WB analysis using anti-myc and anti-actin Abs, respectively. Representative results are shown.

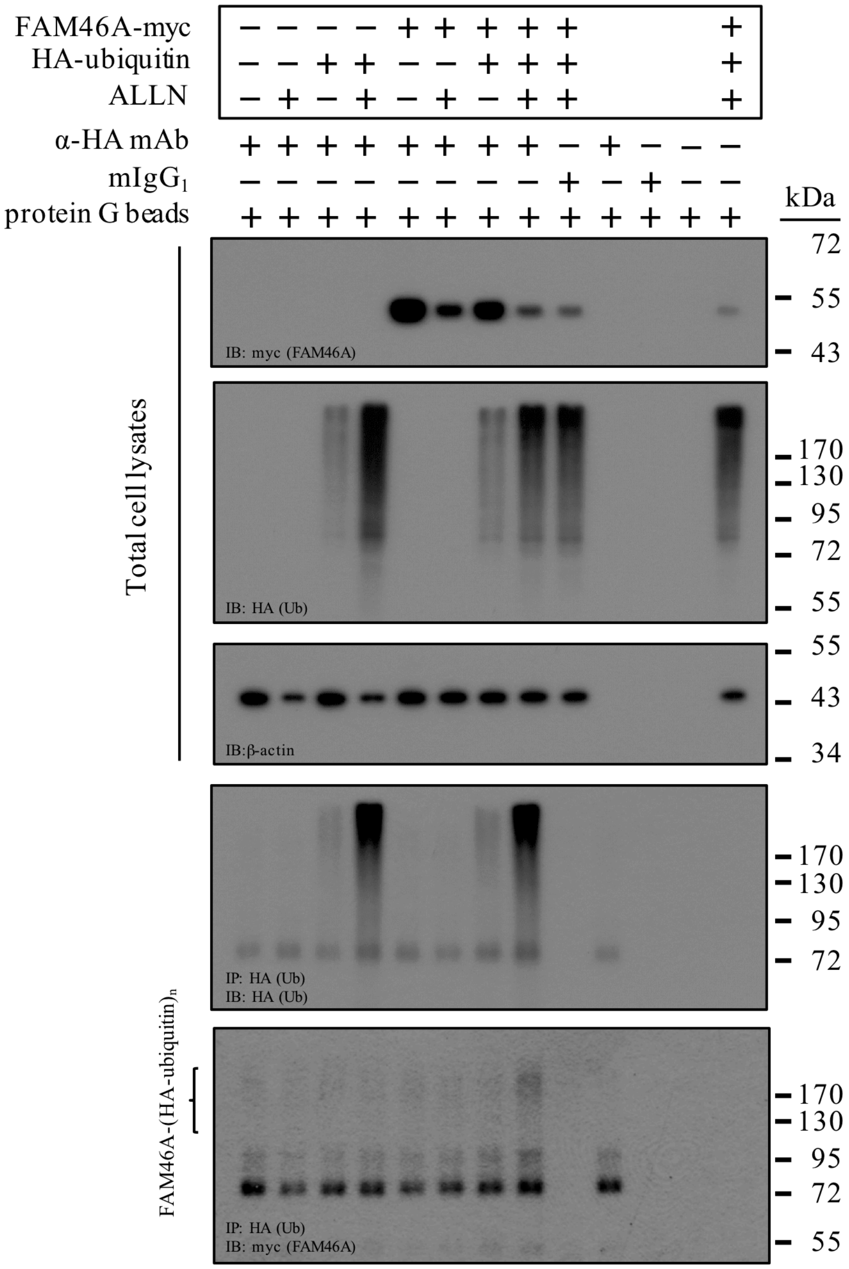

Supplemental Fig. 4 FAM46A is modified by poly-ubiquitylation. HeLa cells were co-transfected with the FAM46A-myc and HA-tagged ubiquitin expression constructs as indicated. Cells were treated with either DMSO (-) or 100 μM ALLN (+) for 16 hr. Whole cell lysates were subjected to immunoprecipitation using anti-HA mAb and protein-G agarose beads. Blots were probed with the anti-myc and anti-HA (clone 12CA5) Abs to detect FAM46A and ubiquitylated proteins, respectively. Expression of β-actin was included as a loading control.

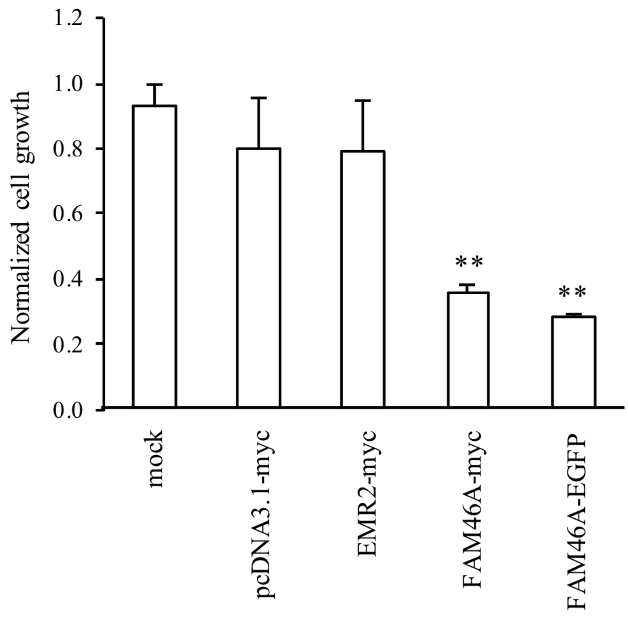

Supplemental Fig. 5 FAM46A expression leads to decreased cell proliferation. HeLa cells were transiently transfected with various expression constructs as indicated. Number of viable cells was examined using the colorimetric CellTiter One Solution assay and recorded as absorbance at 490nm. Normalized cell growth for each group was expressed as an increase in absorbance recorded between 2 hours and 48 hours post transfection. Background absorbance (medium only) was subtracted from these data. Each value represents the mean ± SD of 18 replicates collected from three independent experiments. $^{**}p<0.01$
